# Factors influencing persistence of a threatened amphibian in restored wetlands despite severe population decline during climate change driven weather extremes

**DOI:** 10.1007/s10531-022-02387-9

**Published:** 2022-03-03

**Authors:** Chad T. Beranek, Samantha Sanders, John Clulow, Michael Mahony

**Affiliations:** 1 Conservation Science Research Group, School of Environmental and life Sciences, Biology Building, University of Newcastle, University Drive, 2308 Callaghan, NSW Australia; 2 FAUNA Research Alliance, PO Box 5092, 2290 Kahibah, NSW Australia

**Keywords:** Population persistence, *Litoria aurea*, Reproductive ecology, Drought, Heatwave, Wetland vegetation, Salinity, Chytrid

## Abstract

Biodiversity is in global decline during the Anthropocene. Declines have been caused by multiple factors, such as habitat removal, invasive species, and disease, which are often targets for conservation management. However, conservation interventions are under threat from climate change induced weather extremes. Weather extremes are becoming more frequent and devastating and an example of this was the 2019/2020 Australian drought and mega-fires. We provide a case study the impacts of these extreme weather events had on a population of the threatened frog *Litoria aurea* that occurs in a constructed habitat which was designed to reduce the impact of introduced fish and chytrid-induced disease. We aimed to determine what factors influenced persistence so that the design of wetlands can be further optimised to future-proof threatened amphibians. We achieved this with 4 years (2016–2020) of intensive capture–recapture surveys during austral spring and summer across nine wetlands (*n* = 94 repeat surveys). As hypothesized, drought caused a sharp reduction in population size, but persistence was achieved. The most parsimonious predictor of survival was an interaction between maximum air temperature and rainfall, indicating that weather extremes likely caused the decline. Survival was positively correlated with wetland vegetation coverage, positing this is an important feature to target to enhance resilience in wetland restoration programs. Additionally, the benefits obtained from measures to reduce chytrid prevalence were not compromised during drought, as there was a positive correlation between salinity and survival. We emphasize that many species may not be able to persist under worse extreme weather scenarios. Despite the potential for habitat augmentation to buffer effects of extreme weather, global action on climate change is needed to reduce extinction risk.

**Supplementary Information:**

The online version contains supplementary material available at 10.1007/s10531-022-02387-9.

## Introduction

Many factors have collectively contributed to destabilising biodiversity in the Anthropocene (Houlahan et al. 2000). The Anthropocene has been defined as the period where rapid expansion of human populations has occurred, leading to pronounced impacts on nature due to modification of the environment (Lewis and Maslin 2015). These modifications have been demonstrated to alter and impact populations at various scales (Blaustein et al. 2011). Primary causal impacts include degradation and removal of habitat (Hamer and McDonnell 2008, Arntzen et al. 2017), introduction of invasive species (Kats and Ferrer 2003), introduction of disease (Scheele et al. 2019), and climate change induced weather extremes (Li et al. 2013). The most potentially devastating out of all these primary drivers is anthropogenic climate change, which threatens all life on Earth.

The increasing variations of precipitation due to ongoing climate change increases the frequency of severe weather events such as heatwaves, drought, and wildfire (Trenberth 2011). There have been several severe droughts caused by weather extremes recently in various regions of Earth (Williams et al. 2015, Hoy et al. 2017, McCarthy et al. 2019). Most of these have been identified as being driven by climate change. A recent example was witnessed with the unprecedented drought caused by weather extremes that affected most of Australia during 2019 and 2020, which led to large-scale wildfires across the continent (Nolan et al. 2020).

Biodiversity can be impacted when weather extremes surpass physiological thresholds (Maxwell et al. 2019). Extreme weather has resulted in population declines and local extinctions in many taxa globally (Welbergen et al. 2008, Cahill et al. 2013), from soil microarthropods (Lindberg et al. 2002) to birds of the Southern Great Plains of North America (Cady et al. 2019). Furthermore, the interaction of other threatening processes may be amplified by weather extremes. For example, increased stress caused by severe weather events may result in koalas (*Phascolarctos cinereus*) being more susceptible to disease, which can lead to higher mortality rates and reduced fecundity in diseased females, which results in steeper population decline (Reckless et al. 2018).

Increased drought and heatwaves have severe consequences for amphibians which are dependent on moisture and temperature for their survival. The impacts of drought can be influenced by habitat characteristics and species-dependant responses (Scheele et al. 2012, Clemann et al. 2013, Hossack et al. 2013, Anderson et al. 2015, Zylstra et al. 2019). Drought and heatwaves lead to a net-reduction in amphibian diversity due to local extinction of drought intolerant species and persistence of pre-adapted species (Blaustein et al. 2010, Wassens et al. 2013). Heatwaves can rapidly dry ephemeral wetlands, which can result in mass mortality of tadpoles (Amburgey et al. 2012, Beranek et al. 2020a) and lead to population decline due to lack of recruitment (Weinbach et al. 2018, Swartz et al. 2019). However, some amphibian communities can persist in reduced occurrence during weather extremes and rapidly recolonise wetlands in favourable conditions (Moss et al. 2021).

Amphibian populations are susceptible to threats beyond severe weather events, including habitat loss, disease, and invasive species (Grant et al. 2016). One of the greatest threats is chytrid-induced disease caused by the fungal pathogens *Batrachochytrium dendrobatidis* and *B. salomandrens* which have decimated amphibian populations’ worldwide (Scheele et al. 2019). The severity of on-going habitat loss for amphibian populations applies additional pressure to the conservation status of numerous species (Arntzen et al. 2017).

Restoration and construction of breeding habitat, usually wetlands, is a commonplace strategy to combat the decline of amphibians (Rannap et al. 2009, Magnus and Rannap 2019, Beranek et al. 2020b). The designs of wetlands usually incorporate optimum conditions for breeding to enhance recruitment. Adaptive management may occur to increase breeding site suitability, through actions such as removing introduced predators and reducing wetland vegetation cover (O’Meara and Darcovich 2015, Pollard et al. 2017). More recently, amphibian wetland creation projects have incorporated designs to passively mitigate impacts from threats such as invasive species and chytrid-induced disease to increase survival and recruitment (Beranek et al. 2021c).

It is unknown how constructed wetlands that have increased salinity for chytrid-mitigation and active management of wetland vegetation interplay with drought and heatwaves regarding survival and breeding of target amphibians. It is possible that increased evaporation due to heatwaves could increase salinity levels above thresholds tolerated by amphibians which may result in reduced survival. Since wetland vegetation forms a refuge during unsuitable weather conditions (Clemann et al. 2013), the benefits obtained from enhancing breeding habitat suitability may not be balanced with the potential negative consequences to survival during extreme weather events.

We use intensive capture-recapture of the threatened green and golden bell frog (*Litoria aurea*) in a wetland complex that was constructed to passively mitigate disease and introduced fish, to gain evidence if this habitat design would allow population persistence during extreme weather events. We aimed to (1) determine the impact of drought on the population, (2) determine if weather or habitat influenced survival and (3) identify if breeding occurred on the break of the drought, indicating persistence of the population. We expected that the population would persist but decline significantly during the drought and extreme weather events. We hypothesised that an interaction with the two weather variables that characterise drought; rainfall, and air temperature, would be the strongest predictor of survival. We also hypothesised that increased wetland vegetation cover would be positively correlated with survival, providing evidence that this is an important habitat refuge from extreme weather events. Lastly, we predicted a reduction in survival in response to increased salinity of the wetlands, as we considered that the protective benefits from disease due to increased salinity would be outweighed by the reduced freshwater availability during heatwaves.

## Methods

### Case study species

*L. aurea* is a threatened frog species that occurs in the south-eastern coast of Australia, where its entire distribution was impacted by either drought or wildfire during the 2019–2020 Australian mega-fires (Nolan et al. 2020). The three primary threatening processes considered for this species is chytrid-induced disease (Stockwell et al. 2010), displacement by invasive species (Klop-Toker et al. 2017) and habitat removal (White and Pyke 2008). This species may be vulnerable to impacts of drought (Osborne et al. 2008), however a detailed analysis testing this idea has not yet been undertaken.

Life history traits of *L. aure*a that may mediate the impacts of prolonged drought include *r*-type life history, which is typified by high reproductive output (Gould et al. 2022), short life span and stochastic population fluctuations (Hamer and Mahony 2007, Pickett et al. 2014). Further, based on these traits and coupled with impacts of disease, some populations may only persist with ongoing yearly recruitment (Beranek et al. 2021c). Therefore, *L. aurea* may be more affected by drought episodes that span multiple years. However, Bower et al. (2013) suggested that *L. aurea* may be able to cope with impacts arising from smaller population sizes due to the behaviour of males to form chorus aggregations. This allows females to access several potential mates despite low population sizes (Bower et al. 2013). It is unknown if this behaviour can aid in population persistence after mortality from drought and heatwaves.

### Study site

**Fig. 1:**
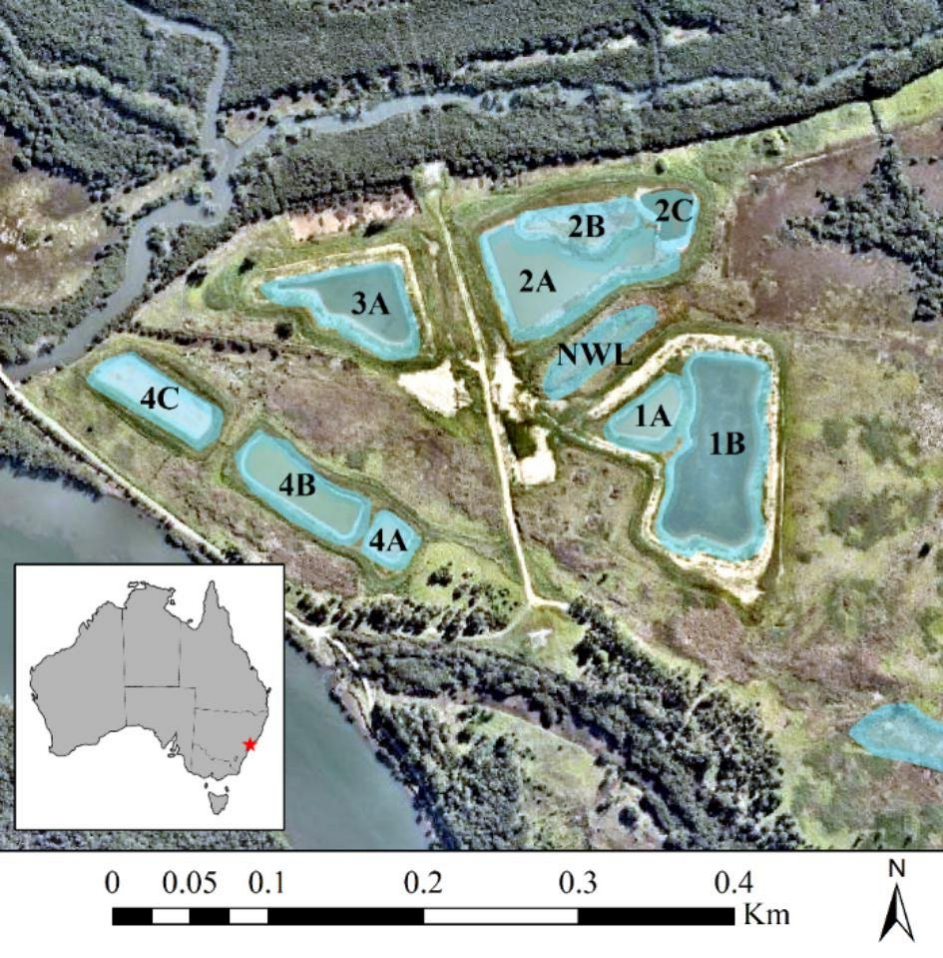
Map of study site. Blue shading indicates wetland extent. Aerial image acquired from Nearmap (image taken on 05/04/2016)

The study site was located on Kooragang Island (Fig. 1) (32°50–54’S, 151°42–47’E) situated ~ 14 km north-west of Newcastle, NSW, Australia. Kooragang Island has had ongoing conservation management of *L. aurea* for over two decades (Klop-Toker et al. 2021). This specific site contains nine created wetlands that were made specifically to enhance *L. aurea* populations. The wetlands were constructed in 2015 and consist of two permanent wetlands (1B and 3A) and seven ephemeral wetlands (1A, 2A, 2B, 2C, 4A, 4B and 4C) that have different hydro-periods. There is also an additional natural wetland within the site (NWL) that supported a small *L. aurea* population before the construction of the created wetlands and it is assumed that individuals of this population colonised the created wetlands when they were constructed. These wetlands also provide habitat for several other threatened wetland fauna (Beranek 2020b, a, Beranek et al. 2021e). See Beranek et al. (2020b) and (Beranek et al. 2021c) for more detailed descriptions of the site.

### Weather events

The monitoring period encompassed the severe drought and mega-fire event that emerged in Australia during 2019 and 2020. In the lead up to the mega-fire, many areas in south-eastern Australia had the lowest volumes of rainfall in the 6 months period before November 2019 ever recorded, coupled with most areas in south-eastern Australia exceeding daily maximum temperatures records by December 2019, creating dry conditions that promoted severe and widespread wildfire (Nolan et al. 2020, Abram et al. 2021). These events have been designated as unprecedented in severity (Bureau of Meteorology 2020, Nolan et al. 2020, Abram et al. 2021).

**Fig. 2:**
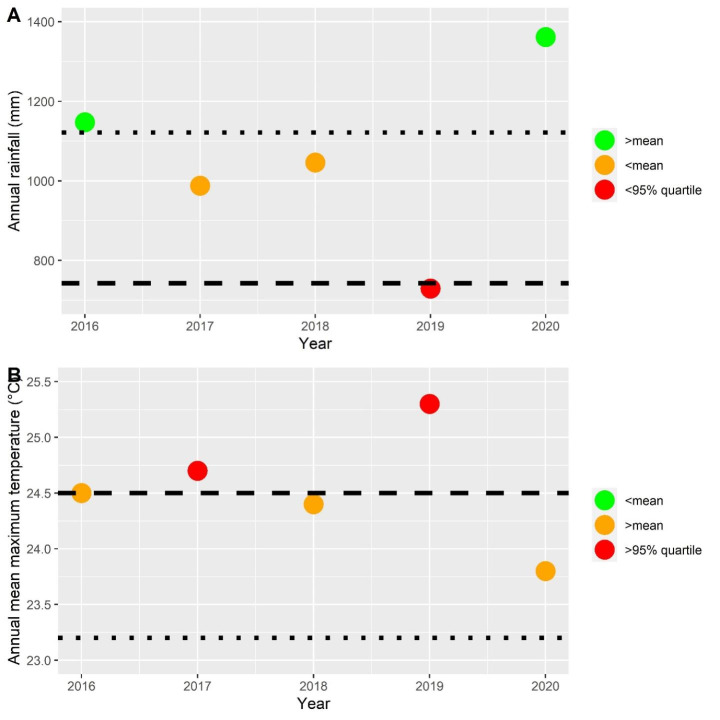
Weather summary across the study period. 2A. Annual rainfall. 2B. Annual mean maximum temperature. Spotted lines indicate the mean, dashed lines indicate the 5th and 95th percentiles for rainfall and temperature respectively. Weather information obtained from Williamtown RAAF, station number: 061078

The most representative weather station of the field site (Williamtown RAAF, station number: 061078, located ~ 13.8 km northeast of the study site, data from the Bureau of Meteorology 2020), recorded annual rainfall measurements that were in the 5% lowest percentile, and this was the third lowest recording at this station dating back to 1944 (729.4 ml, see Fig. 2A). There were no summer/early autumn rainfall events recorded in 2019 that were large enough to replenish the wetlands, yet this occurred in all subsequent years of the study (see S1 for details of hydrology in the wetlands). In terms of replenishment, the wetlands did not reach full charge from December 2018 – February 2020. This area experienced a ~ 14-month drought.

The mean annual maximum temperature for 2019 was the hottest on record (25.32 °C) and was warmer by > 0.6 °C compared to subsequent years (2016 = 24.5 °C, 2017 = 24.66 °C and 2018 = 24.33 °C, see Fig. 2B). Two months in 2019 at the Williamstown RAAF station set mean maximum temperature records (January and November), while every other month had mean maximum temperatures about 2 °C above the monthly mean.

### Amphibian surveys

Capture-recapture surveys were conducted by walking uniformly in gridded transects that encompassed the entire surface area of the wetlands and adjacent terrestrial areas contained within each site (~ 5 m from the bank). Frogs were detected using head-torches (LED Lensor 7.2R and 14.2R). Frogs were captured by hand and placed in single use clean plastic bags that were labelled with a unique collector-coded number, GPS location, and time. All bagged frogs were brought to a central processing point where details on sex, size and tag number were obtained. A microchip reader was used to determine whether the frog had been caught previously and had a tag (passive integrated transponder tag - PIT tag). If the frog did not have a tag and was > 30 mm snout-vent length (SVL), a tag (Trovan midi-chip) was inserted under the skin as described by Christy (1996). After processing, frogs were released to the precise point of capture. The time between capture and release could be up to 1 h.

There was a total of 94 capture-recapture surveys which were conducted almost weekly from September 2016 to April 2020, restricted to months incorporating the breeding season of *L. aurea* (September—April). The number of weekly capture recapture surveys for each breeding season were 28 for 2016/2017 (year 1), 24 for 2017/2018 (year 2), 26 for 2018/2019 (year 3) and 16 for 2019/2020 (year 4). The latter season had a smaller sample size due to safety restrictions that were in place from mid-December 2019—mid-January 2020 due to bushfire risk, and then more restrictions were put in place during April 2020 due to COVID-19. The natural wetland NWL was surveyed four times each season and frogs captured at this site were not included in CMR analysis.

The number of ‘breeding events’ were estimated based on unique observations of eggs, tadpoles and metamorphs. Hence, a ‘breeding event’ was defined as an instance of discrete breeding that occurred in a wetland, as evidenced by either an observation of an egg clutch, or a cohort of tadpoles/metamorphs. Observations of breeding events were made during capture-recapture surveys. More details of the breeding events are reported in (Beranek et al. 2021d).

### Statistical analyses

Capture-recapture histories were constructed for each adult frog. The data was pooled across all wetlands as individuals freely and often move between them. We opted to use an open population model, the POPAN formulation of the Jolly-Seber model (hereafter referred to as POPAN, Schwarz and Arnason 1996). We used this model since *L. aurea* has traits that are typical of an *r*-type strategist including a high recruitment rate and low survival rate (Hamer and Mahony 2007, Pickett et al. 2014, Gould et al. 2020). Therefore, an open model is appropriate because it can relax the assumptions of no death/recruitment or immigration/emigration between capture periods. In addition, POPAN has been used in other studies of this species and the estimates are comparable to the robust models, also used in past studies for this species (Pickett et al. 2014, Goldingay et al. 2017).

The parameters in POPAN are φ apparent survival probability, *P* detection probability, given the individual is present within the study area to be captured, *pent* the probability of an individual being available to be caught, and N the size of the super-population. φ is classed as apparent survival as it is not possible to disentangle mortality from permanent emigration. N is defined as the total number of individuals that were present at some time during the period between the first and last sampling occasions. POPAN models allow derived estimates of the population size per survey.

There were several variables used to model the variation in each parameter in the POPAN models (See Table 1). Before covariates were included into models, a Spearman’s correlation matrix was created to assess collinearity between all covariates. Covariates that were found to have a correlation of |>|0.4 were deemed collinear and were not included in the same model (see S2). Salinity and mean maximum air temperature were found to be collinear and were not analysed together in models. Before covariates were included into models, they were standardised to Z-scores by subtracting each value from the mean and dividing by the standard deviation.

Covariates were included into each parameter as standalone submodels. First, sex was included as a group parameter in N for all models. Second, *P* was fitted with 30 combinations of different covariate combinations where the most parsimonious inclusion was carried for inclusion into the pent submodel. The parameter pent was fixed with an interaction between sex and year since a previous investigation of this population demonstrated that adult recruitment for each sex varied in each year due to unequal maturation times (Beranek et al. 2021b). It was found that the model could not converge with this pent parameterization due to the estimates in the fourth year for each sex being at a limit of estimation. Hence, we fixed pent for year four in both sexes to 0 recruitment. This was deemed valid as late season summer breeding events did not occur in year 4 and it has been demonstrated that these events produce the greatest recruitment of *L. aurea* metamorphs (Beranek et al. 2021d). Finally, the various hypotheses of φ were tested in final submodels, with the null model being φ(.), *P*(time), pent(Sex*year), N(Sex). The Akaike Information Criteria (AIC) was used to determine the most parsimonious models.

**Table 1:** Co-variates tested against each parameter. ^1^ The weather data was obtained from the Bureau of Meteorology, Williamtown RAAF NSW weather station (number: 061078)

Parameters	Variable	Description
*P*	effort	The total number of person minutes occurring during the survey occasion.
*P*	time	Variability between each capture occasion.
φ	mmaxt	Mean previous maximum temperature (°C) dependant variability. Measured as the mean maximum temperature in the previous 5-days before the survey occasion.^1^
*P*	maxt	Maximum temperature (°C) dependant variability. Maximum temperature of the day surveys occurred on.^1^
φ, *P*	rain	Rain dependant variability. Measured as the amount of rainfall (mm) in the previous 5-days before the survey occasion.^1^
φ	sal	Mean salinity of all the wetlands combined (ppt).
*P*, pent	sex	Categorical individual variable, 1 = female and 0 = male.
φ	veg	Vegetation cover of percentage of all the wetlands combined. Expressed as a proportion.
pent	year	Categorical variable for the breeding season the survey was conducted in. Consisted of year 1 = 2016–2017, year 2 = 2017–2018, year 3 = 2018–2019 and year 4 = 2019–2020.

The submodel for survival was parameterized to test the following hypotheses regarding correlates of *L. aurea* survival:

#### Hypothesis 0

Null (survival constant across time).

#### Hypothesis 1

Mean maximum air temperature.

#### Hypothesis 2

Rainfall.

#### Hypothesis 3

Salinity of wetlands.

#### Hypothesis 4

Vegetation cover.

#### Hypothesis 5

Mean maximum air temperature and rainfall.

#### Hypothesis 6

Mean maximum air temperature and vegetation cover.

#### Hypothesis 7

Rainfall and salinity of wetlands.

#### Hypothesis 8

Rainfall and vegetation cover.

#### Hypothesis 9

Salinity of wetlands and vegetation cover.

#### Hypothesis 10

Mean maximum air temperature, rainfall, and vegetation cover.

#### Hypothesis 11

Salinity of wetlands, rainfall, and vegetation cover.

#### Hypothesis 12

Interaction between rainfall and vegetation.

#### Hypothesis 13

Interaction between rainfall and salinity of wetlands.

#### Hypothesis 14

Interaction between vegetation cover and salinity of wetlands.

#### Hypothesis 15

Interaction between mean maximum air temperature and vegetation cover.

#### Hypothesis 16

Interaction between rainfall and mean maximum air temperature.

Derived weekly population estimates were obtained from the highest performing model to visualise weekly population fluctuations before and during the drought and to determine the adult population size for males and females and at the time the drought was broken. This was achieved by using the popan.derived function in *RMark*. For every population estimate, the error is reported as 95% confidence intervals (95% CI).

Over-dispersion was assessed by using a goodness of fit test with the release.gof function in *RMark*. This allowed an estimation of the ĉ-hat over-dispersion parameter. If ĉ-hat was < 2, the models were deemed not over-dispersed, and no adjustments were made. If ĉ-hat was > 2, the models were deemed over-dispersed and adjustments to the models were made using the ĉ-hat value in the function adjust.chat.

### Genetic analysis

Genetic collection and analysis were performed as per Beranek et al. (2021a), and here we provide a summary of this method. DNA extraction and sequencing was conducted by Diversity Arrays Technology (DArT PL). Its patented next generation sequencing protocol, DArTseq, is a cost-effective option for generating high quality, high-throughput SNP datasets for non-model species. A description of the DArTseq protocol is available in Jaccoud et al. (2001). From this, 41,151 loci were produced. The loci were filtered according to a call rate of 100% (loci remaining, hereafter lr = 13,733), reproducibility of 100% (lr = 5,096), minor allele frequency of 5% (lr = 2,677), removal of secondaries (lr = 2,671) and a linage disequilibrium of 90% (lr = 2,492). All filtering steps were conducted in R statistics using the package DArTR (Gruber et al. 2018). The remaining loci were considered high quality for the purposes of relationship assignment.

To determine the amount of mating pairs that were produced in breeding events in the break of the drought, genetic pedigree analysis was conducted on a sample of eleven tadpoles derived from a single wetland (2A). COLONY version 2 (Jones and Wang 2010) was used to identify relationships between adult frogs (*n* = 15 females and 8 males) and tadpoles to assign maternity and paternity (*n* = 11), and between tadpoles, to screen for full-siblingship, half-siblingship, or unrelated pairings.

A Monte Carlo simulation was conducted to estimate the number of *L. aurea* eggs produced within the wetland where genetics was obtained, determined by how many maternity clusters were identified. The simulation assumed a normal distribution, where sum of 8 simulated egg clutches was taken from a mean and standard deviation derived from egg counts of *L. aurea* provided by (van de Mortel and Goldingay 1998, mean = 5706, SD = 507 and *n* = 8). This was repeated with 100,000 iterations to estimate the mean and 95% confidence intervals.

## Results

**Fig. 3:**
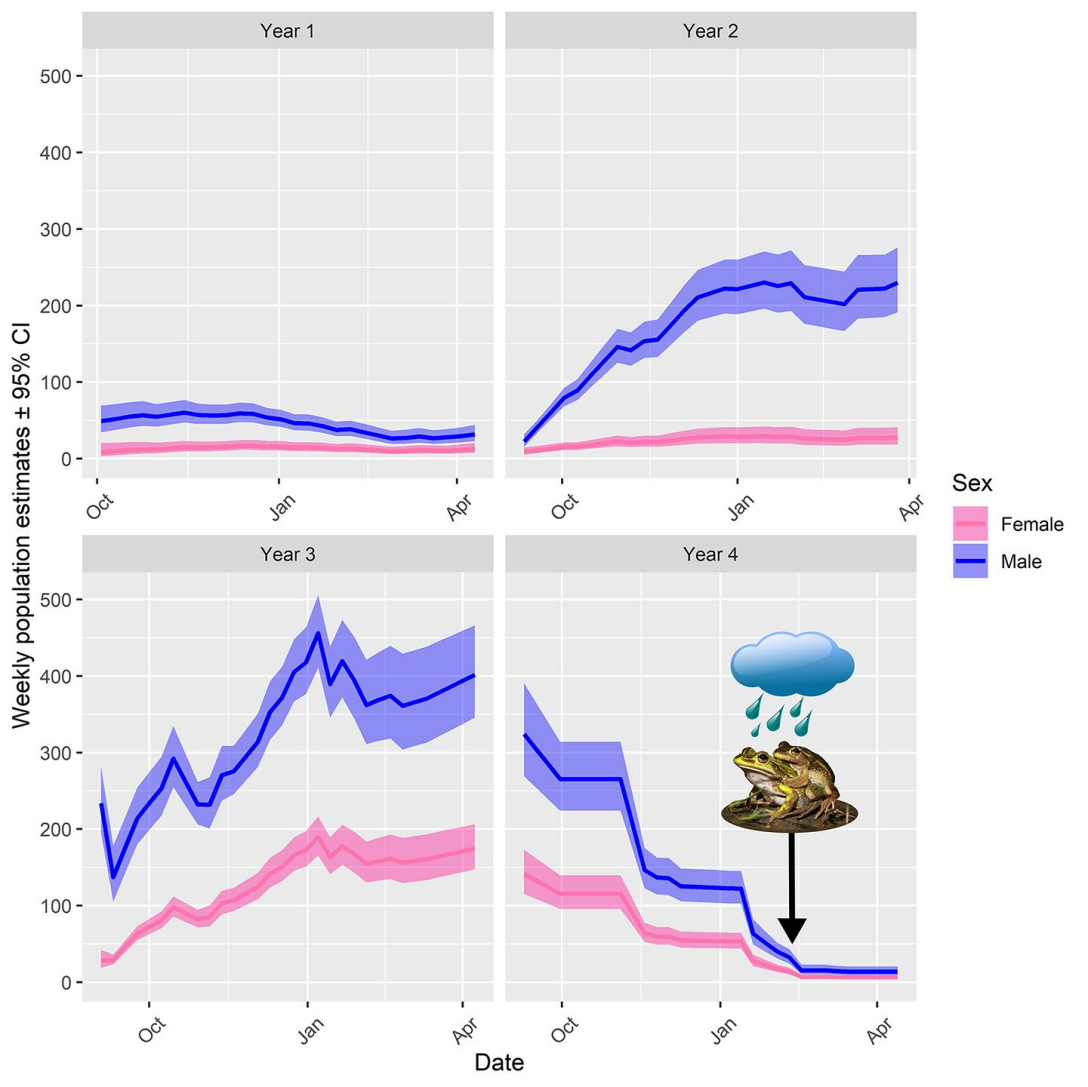
Derived weekly adult population estimates of *Litoria aurea* during the 2019–2020 breeding season (± 95% CI). Results obtained from the top performing model (H16). *n* = 94 repeat capture-recapture surveys. Weeks where surveys did not occur have been interpolated. Arrow indicates the week where the drought was broken, and breeding occurred

Overall, 745 adult males and 242 adult females of *L. aurea* were captured over the four-year period. From these individuals, 1,069 male and 301 female captures were made. The maximum number of times an individual male was captured was 15, whereas the maximum for a female was 10 (see S3 for yearly summary of captures). There was a general increase in adult *L. aurea* captures in first three years and a decrease in the fourth year (see Beranek et al. 2021b, c for comprehensive analyses of population dynamics in the years before the drought). Population size fell sharply for both sexes throughout fourth season (see Fig. 3). The sharpest weekly declines for both sexes occurred in November 2019 and January 2020. In both months, the decline of males lagged the decline of females.

**Fig. 4:**
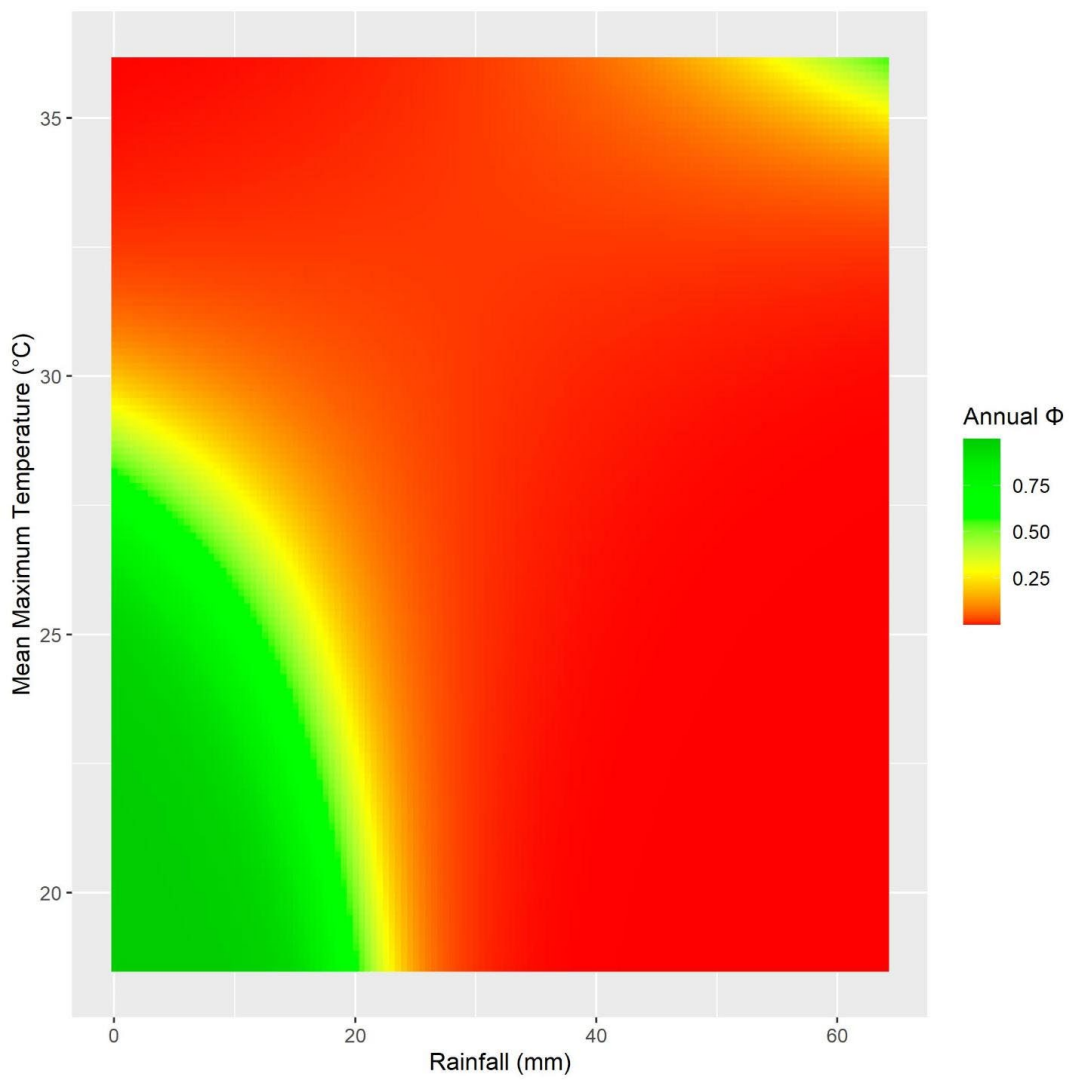
Interaction heatmap between rainfall and mean maximum temperature and their effects on annual survival probability. Rainfall = total rainfall in the previous 5 days. Mean maximum temperature = the mean maximum temperature in the previous 5 days. Results obtained from estimates in the top performing model (H16)

There was support for all four variables, rain, mmaxt, veg and sal, being important predictors of φ (see Table 2). The most parsimonious model included an interaction between rain and mmaxt (see Fig. 4). Vegetation cover was featured in the next nine top models and retained a significant positive relationship with φ in seven models. Mean salinity was included in two of the top five models, both instances in models that also contained vegetation coverage. Mean salinity retained a significant positive relationship with φ in all models it was included in. The effect of mean rainfall was significant in most models except when combined in models with vegetation cover. Mean maximum temperature was not a strong predictor of φ in most models except for when included as an interaction with rain.

**Table 2:** Comparison of hypotheses explaining survival. *n* = number of parameters included in model. H = hypothesis, df = degrees of freedom. *β* coefficient estimates are reported for each variable in the model with ± 95% confidence intervals reported in brackets

H	φ (intercept)	φ (rain)	φ (mmaxt)	φ (veg)	φ (sal)	φ interaction	df	ΔAIC
H16	**-0.96 (-1.66,-0.25)**	**-3.28 (-4.71,-1.84)**	**-4.90 (-6.53,-3.27)**			**5.53 (3.98,7.08)**	106	0
H14	2.88 (-11.54,17.31)			-19.46 (-44.18,5.26)		10.21 (-0.38,20.79)	106	7.59
H10	**-9.65 (-13.77,-5.53)**	-0.77 (-1.69,0.15)		**3.78 (1.56,5.99)**	**2.21 (0.58,3.84)**		106	8.92
H6	**-9.71 (-13.76,-5.65)**			**4.50 (2.39,6.61)**	**2.14 (0.55,3.72)**		105	9.43
H12	**-3.86 (-5.46,-2.27)**	2.87 (-3.68,9.41)		2.52 (-0.56,5.60)		-7.01 (-18.85,4.82)	106	10.50
H7	**-4.26 (-5.34,-3.18)**	-0.96 (-1.96,0.04)		**3.41 (1.37, 5.45)**			105	11.23
H3	**-4.47 (-5.57,-3.37)**			**4.24 (2.30, 6.18)**			104	12.51
H11	**-4.37 (-5.49,-3.25)**	-1.22 (-2.48,0.04)	-0.35 (-1.34,0.65)	**3.45 (1.42,5.47)**			106	13.01
H5	**-4.38 (-5.54,-3.22)**		0.20 (-0.62,1.02)	**4.12 (2.11,6.12)**			105	14.55
H15	**-4.61 (-5.84,-3.38)**		-0.94 (-2.93,1.06)	**4.53 (2.40,6.67)**		-1.06 (-1.49,6.45)	106	15.29
H13	**-7.95 (-12.46,-3.45)**	0.63 (-1.46,2.72)			**2.26 (0.30,4.22)**	**-1.00 (-1.81,-0.20)**	106	19.96
H9	**-7.53 (-11.31,-3.75)**	**-1.53 (-2.41,-0.65)**			**2.10 (0.46,3.74)**		105	19.96
H1	**-2.59 (-2.90,-2.27)**	**-1.75 (-2.67,-0.83)**					104	21.68
H8	**-2.64 (-3.06,-2.23)**	**-1.89 (-3.08,-0.70)**	-0.18 (-1.12,0.76)				105	23.79
H4	**-6.88 (-9.98,-3.78)**				**1.97 (0.64,3.31)**		104	30.25
H2	**-2.06 (-2.31,-1.81)**		**0.79 (0.01,1.58)**				104	32.17
H0	**-2.16 (-2.37,-1.96)**						103	34.19

### Breeding summary

There were 37 breeding events detected over the four-year period. These occurred in distinct time periods, either in September – December and/or February – April. There was breeding representing both these periods in all years, except no late season breeding was detected in year 3 (i.e., from Feb – Apr), as there was not sufficient rainfall to replenish wetlands. See Beranek et al. (2021d) for more details.

Breeding during the break of the drought in 2020 was predicted to have taken place on the 9/02/2020, as a chorus aggregation of ~ 10 males were observed in wetland NWL during the day on the 10/02/2020, and subsequent tadpoles were observed in 2 A and NWL. The adult population sizes for the week when the drought was broken was 14 (11–19 95% CI) and 40 (31–52 95% CI) for females and males respectively (see Fig. 3).

From the eleven randomly sampled metamorphs from 2 A, there were three full-sibling groups. There was one female parent identified for tadpoles 0037, 0039, 0040 and 0036 (probability of maternity assigned for all offspring = 1.00), despite half-siblingship identified between 0036 and 0037, 0036–0039, 0036–0040, 0040 − 0037 and 0040–0039 (probability of half-siblingship for all pairings = 1.00). There was one other full-sibling pairing identified, 0032–0030 and 0035–0038 (probability of full-siblingship for both pairings = 1.00). There were no other instances of half-siblings being assigned.

Given that there were seven kinship groups in wetland 2 A, and that at least one kinship group was present in NWL as there were tadpoles and metamorphs observed in this wetland, at least eight females reproduced during this breeding event. This equates to 57% (from 42 − 73%) of all adult females in the estimated extant population as identified from derived weekly estimates. It is estimated that 45,648 eggs (42,839 − 48,459 95% CI) were produced in 2 A on the break of the drought.

### Juvenile colonisation

In March 2020, there were 296 and 61 metamorphs recorded in created wetland 2A and natural wetland NWL respectively. In April 2020, most had achieved metamorphosis (the remaining metamorphs *n* = 3 in 2A, *n* = 0 in NWL) and had colonised all the surrounding wetlands that contained water. A total count of the juvenile frogs observed across all wetlands was 319 (1A *n* = 39. 1B *n* = 2, 2A *n* = 43, 2B *n* = 2, 2C *n* = 2, 3A *n* = 216, 4A *n* = 8, 4B *n* = 7, 4C and NWL *n* = 0). There were an additional six juveniles detected opportunistically in terrestrial habitat.

## Discussion

We demonstrate that the unprecedented weather extremes caused severe decline in a threatened amphibian in wetlands constructed as a refuge from other threatening processes. There are two population ecology processes that led to the observed drop in population size: (1) reduction in recruitment and (2) high mortality rates. As predicted, the most parsimonious model included both maximum temperature and rainfall as an interaction on survival, indicating that the unprecedented warm air temperatures coupled with low rainfall led to a reduction in survival. Despite these impacts, we provide evidence that *L. aurea* is resistant to potential impacts of small population sizes, as breeding and subsequent juvenile recolonization occurred and hence, we identify this species as having some resilience to severe weather impacts. As hypothesised, we found evidence that wetland vegetation provided a buffer from weather extremes. Against our hypothesis, we found that salinity in the created wetlands was positively correlated with survival indicating that this parameter did not add further stress to the population during the drought. We provide discussion on implications for conservation management and restoration of habitat for threatened species, with emphasis on chytrid impacted amphibians, in response to climate-change induced weather extremes.

### Causes of population declines

The rapid decline in the adult population size during the drought year was likely influenced by lack of adult recruitment due to a missed late summer breeding opportunity in the previous season. Since *L. aurea* produce ~ 8.2-fold more metamorphs in late season breeding compared to early season breeding (Beranek et al. 2021d), it is likely that late summer breeding events in previous seasons modulate contemporary adult population sizes. The already low survival rates in previous seasons in this population acted synergistically with low recruitment to cause the sharp decline (Beranek et al. 2020b). Since numerous species share the fast history-life strategy of *L. aurea*, they may be similarly affected by severe weather extremes (Anderson et al. 2015).

Recruitment in previous breeding seasons has been identified in several other studies as a key determinant of contemporary population sizes (Scheele et al. 2012, Cayuela et al. 2016a). This provides justification that impacts to recruitment should be targeted for understanding declines brought about by climatic anomalies. If there were two seasons where late season breeding did not eventuate due to a lack of summer rainfall, local extinction risk in *L. aurea* would increase due to an absence of recruitment and the ubiquitous threat of high mortality rates. Similar findings point to the impacts of severe weather events on recruitment in other species. For example, during the same heatwave in Australia in 2019, there was a spike in pup abandonment in the grey-headed flying fox (*Pteropus poliocephalus*), where at least 2612 pups died resulting in reduced recruitment for the subsequent generation (Mo et al. 2021).

Our result that amphibian survival was correlated with rain and temperature agrees with other studies (Cayuela et al. 2016a, Cayuela et al. 2016b) and demonstrates an impact of the drought. The lack of rain and high temperatures likely contributed to mortality in *L. aurea* during the drought. Dehydration can cause severe impacts to the physiology of amphibians, affecting the capacity for movement and aerobically supported activities (Hillman et al. 2008). High temperatures may cross a threshold that results in sudden death of amphibians due to nerve shutdown (von May et al. 2019). This threshold is known as the thermal maxima (CT_max_). However, *L. aurea* appears to have physiological mechanisms that may permit it to withstand drought. For example, this species has a water vapour flux capacity that allows it to persist during high temperatures for longer periods of time compared to other similarly sized amphibians (Buttemer 1996). It is important that future studies quantify the CT_max_ in other species so that the consequences of rises in atmospheric temperatures can be predicted.

### Factors influencing persistence during weather extremes

The survival of amphibians that persist in saline disease refugia is balanced between two extremes. During periods of cold and wet weather, their survival can sharply decrease due to a high prevalence of chytrid-induced disease (Sonn et al. 2019). In these situations, salinity of the environment will be diluted which will further promote chytrid growth. On the other hand, if heatwaves are experienced, the environment can dry, and evaporation can cause salinity levels to rise above tolerable thresholds (Clemann et al. 2013). We found that across the study period there was a positive trend between salinity and survival. This provides evidence that the frogs avoided the potentially harmful influence of salinity during the drought and benefited from the enhanced salinity. While *L. aurea* are known to use aquatic refuges (Garnham et al. 2015), it is likely they would leave these microhabitats once salinity levels pushed close to physiologically tolerable thresholds and would relocate to a different microhabitat structure with more suitable properties. Further research is needed in microhabitat selection of chytrid-impacted amphibians to test this hypothesis. We consider the positive correlation of survival and salinity adds weight to the idea that this is a valuable design feature to incorporate into wetland creation projects for chytrid-impacted amphibians to reduce disease and increase survival, despite the impending impacts of climate change.

Wetland vegetation coverage appeared to have a role in promoting survival. This presumably enhanced protection from predators and provided a refuge that buffered temperature extremes (Sinclair et al. 2016). Wetland vegetation is an important drought refuge for the southern bell frog (*Litoria raniformis*), a close relative of *L. aurea* (Clemann et al. 2013). Other forms of vegetation have also been identified as refuges for different species worldwide (Scheffers et al. 2013). Wetland vegetation offers a suitable microclimate during adverse weather conditions, having cooler and more stable temperature than other microhabitats (Garnham et al. 2015) and presumably retains moisture longer, which amphibians are physiologically dependant on. We encourage research that identifies climatic microhabitat refuges in other species worldwide so that habitats can be enhanced to increase the probability of survival.

We recommend practitioners to maintain high coverages of wetland vegetation in existing sites and future wetland designs to buffer potential impacts from climate change induced weather anomalies. This form of management is in-line with suggestions of Shoo et al. (2011) who determined that amphibians can be made more resilient to climate change-induced weather extremes by engineering microclimate refuges that prevent over-heating and desiccation. Management practices for enhancing habitat for *L. aurea* has included removing wetland vegetation (O’Meara and Darcovich 2015). There is currently no conclusive evidence that this promotes breeding activity or enhances occupancy (Fardell et al. 2018). More research is needed to determine if this is beneficial, but we highlight that it is a detrimental action during heatwaves and droughts. We recommend practitioners to cease wetland vegetation removal in the lead up to or during extreme weather anomalies.

While habitat may provide a refuge from weather anomalies, surrounding habitat connectivity is necessary for the persistence of animal populations after these events (Opdam and Wascher 2004, Sitters and Di Stefano 2020). We observed rapid colonisation of surrounding wetlands through juvenile dispersal, highlighting the importance of habitat connectivity in recovering populations after severe climatic events. The importance of maintaining habitat connectivity for amphibians is supported by an experimental study by Cline and Hunter Jr (2016) where juvenile movement in *Lithobates sylvaticus* was influenced by habitat type and this species was more willing to move through vegetation compared to artificial surfaces (Cline and Hunter Jr 2016). This may be paramount in species that have a ‘boom-bust’ ecology like *L. aurea* where local extinctions and recolonisations are common aspects of metapopulation dynamics (Hamer and Mahony 2007, Heard et al. 2012).

Short-term population persistence may have been achieved but it is unknown if breeding would have been successful if the male chorus that occurred on the break of the drought was smaller. There is evidence that chorus size is related to breeding success in the endangered Houston toad (*Bufo houstonensis*) (Gaston et al. 2010). Similarly, male *L. aurea* are known to congregate during chorusing (James et al. 2015), however it is unknown if chorus size is related to breeding success. Future behavioural research is needed to identify if chorus size in wild amphibian populations is correlated with breeding success, as this life-history trait may predict population persistence when there are bottlenecks in population size due to weather extremes.

Global action on climate change is required to reduce the risk of biodiversity loss. It is unknown if *L. aurea* and amphibians with similar life history traits can persist under more extreme drought conditions that have been presented in this article. This is concerning for the conservation of this species as the annual probability of survival for adults of most populations is low under non-drought condition (< 0.44 Pickett et al. 2014), and extreme weather events are predicted to be more prolonged and severe for south-eastern Australia and many other regions of the planet under moderate climate change scenarios (Kirono et al. 2011). Extreme weather anomalies in future climate change scenarios are likely to surpass the critical survival thresholds for many species (Maxwell et al. 2019). Climate change is likely to impact many animal populations globally (Walls et al. 2013, Cayuela et al. 2016a, Potvin et al. 2017, Zylstra et al. 2019). Synchronised global action on climate change is needed to fortify conservation efforts.

In conclusion, we provide evidence that amphibians can persist during unprecedented drought in constructed wetlands. We highlight that extreme weather events can cause severe decreases in adult population size, and reduced recruitment. Despite low population sizes, reproduction was achieved at the break of the drought, which led to recolonization of wetlands through juvenile dispersal. Salinity did not negatively influence survival, which validates this wetland feature for inclusion in future amphibian habitat restoration projects where chytrid mitigation is an objective. Wetland vegetation appeared to have buffered some impacts of the heatwaves and drought. Mitigation of climate change driven weather events for amphibians can be achieved by ensuring wetlands have high coverages of wetland vegetation and by maintaining habitat connectivity for recolonization post-drought. Ongoing research is needed to identify microhabitat refuges that buffer impacts from extreme weather events for other species to aid in conservation management under the dire climate change predictions. However, we reiterate that this is a ‘Band-Aid’ solution without global action to combat climate change.

## Electronic Supplementary Material

Below is the link to the electronic supplementary material.

Supplementary Material 1

## Data Availability

The data is available on Mendeley Data, doi: 10.17632/hx8nz49vvd.1.
