# Supplementary material for: Factors influencing persistence of a threatened amphibian in restored wetlands despite severe population decline during climate change driven weather extremes
Source: Biodivers Conserv. 2022 Mar 3;31(4):1267–87. doi: 10.1007/s10531-022-02387-9 (PMC8893051; doi:10.1007/s10531-022-02387-9)
Supplement: Supplementary file 1 — Supplementary Material 1 [file 10531_2022_2387_MOESM1_ESM.docx]

Supplementary Material: Factors influencing persistence of a threatened amphibian in restored wetlands despite severe population decline during climate change driven weather extremes

**Authors:** Chad T. Beranek^1,2^, Samantha Sanders^1^, John Clulow^1,2^ & Michael Mahony^1^

^1^Conservation Biology Research Group, School of Environmental and life Sciences, Biology Building, University of Newcastle, University Drive, Callaghan NSW 2308, Australia.

^2^FAUNA Research Alliance, PO Box 5092, Kahibah, NSW 2290, Australia

**Corresponding author –** E: [chad.beranek@uon.edu.au](mailto:chad.beranek@uon.edu.au), P: +61 406 769 007

**S1 – Hydrology of the wetlands across each season.**

**Figure 1.** Hydrology in permanent wetlands in year one (2016-2017).

**Figure 2.** Hydrology in ephemeral wetlands in year one (2016-2017).

**Figure 3.** Hydrology in permanent wetlands in year two (2017-2018).

**Figure 4.** Hydrology in ephemeral wetlands in year two (2017-2018).

**Figure 5.** Hydrology in permanent wetlands in year three (2018-2019).

**Figure 6.** Hydrology in ephemeral wetlands in year three (2018-2019).


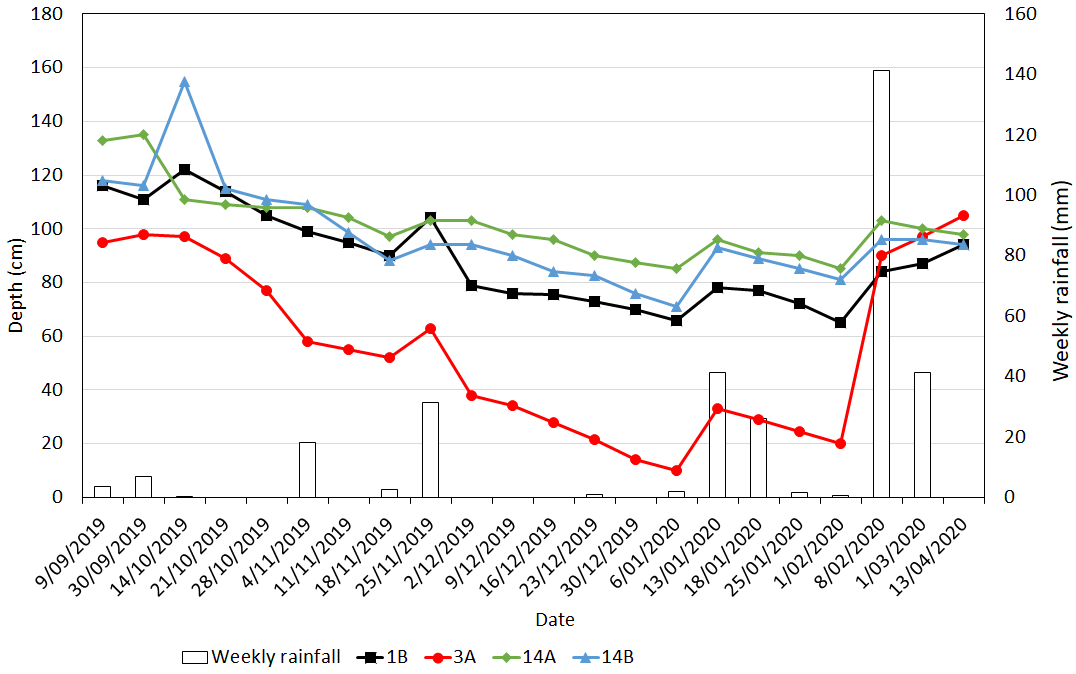


**Figure 7.** Hydrology in permanent wetlands in season four (2019-2020).


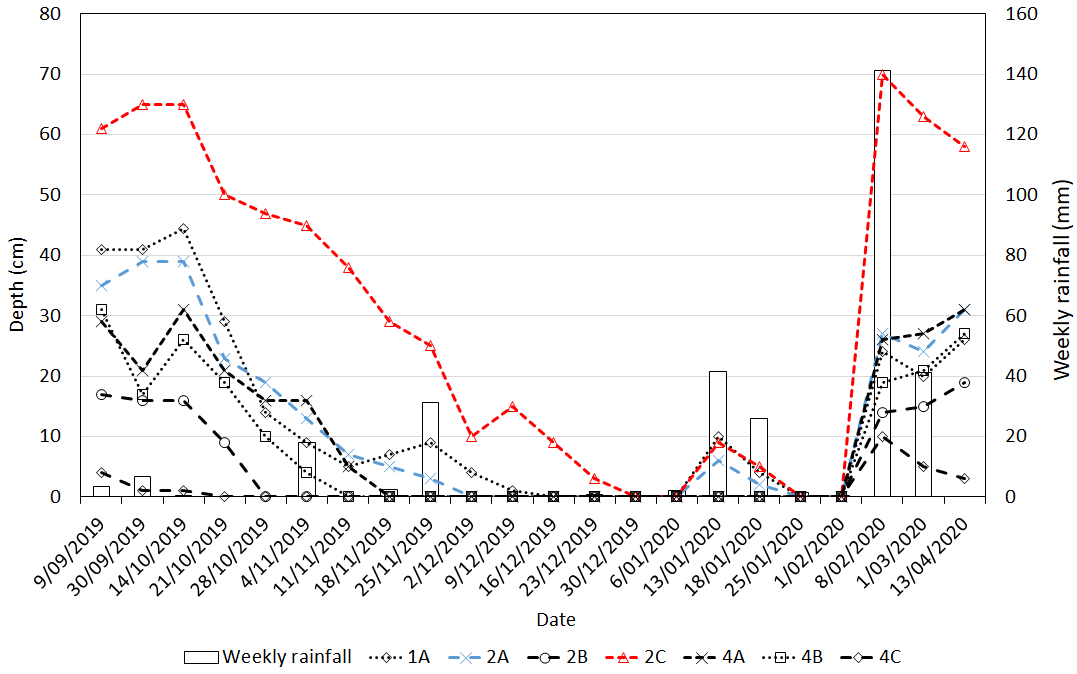


**Figure 8.** Hydrology in ephemeral wetlands in season four (2019-2020).

**S2 – Spearman’s correlation matrices**

**Table 1.** Spearman’s’ correlation matrix of covariates using in probability of detection.

| Covariate | Effort | Rain | Maxt |
| --- | --- | --- | --- |
| Effort | 1.00 |  |  |
| Rain | -0.08 | 1.00 |  |
| Maxt | 0.11 | -0.19 | 1.00 |

**Table 2.** Spearman’s’ correlation matrix of covariates using in probability of detection.

| Covariate | Rain | Mean maxt | Veg | Sal |
| --- | --- | --- | --- | --- |
| Rain | 1.00 |  |  |  |
| Mean maxt | -0.27 | 1.00 |  |  |
| Veg | -0.08 | 0.07 | 1.00 |  |
| Sal | -0.03 | 0.49 | 0.14 | 1.00 |

**S3 – Summary of captures**

**Table 1.** Summary of captures.

| Season | No. males (captures) | No. females (captures) | Total adults (captures) |
| --- | --- | --- | --- |
| 2016-2017 | 89 (213) | 26 (67) | 115 (280) |
| 2017-2018 | 214 (292) | 29 (59) | 243 (351) |
| 2018-2019 | 352 (554) | 121 (173) | 463 (727) |
| 2019-2020 | 158 (248) | 75 (121) | 233 (369) |

**S4 – List of Earthwatch volunteers**

Disappearing Frogs Expedition January 2018 – Earthwatch representative: Andrea Haas, attendees: Chayton Barber, Stella Cross, Aidan Fong, Matthew Harris, Cassandra Ho, Rebecca Jenkins, Elijah Kinnane, Mila Norquay-Whitford, Niza Salarda and Glen Sands

Disappearing Frogs Expedition December 2018 – Earthwatch representative: Andrea Haas, attendees: Emily Bridges, Eirene Carajias, Zara Edmond, Abaigh Gleeson, Abby Howes, Siobhan Kirk, Andrew Lim, Kirralee Seaman, Michela Skipp, Chenxin Tu and Riley Warwick

Disappearing Frogs Expedition January 2019 – Earthwatch representative: Maria Garcia-Rojas, attendees: Rosemary Bergin, Lucy Capurso, Isaac Cheng, Renee Kennedy, Claire Larkin, Victoria Mok, Emma Peterson, Emily Saddington, Anna Tran and Adrian Yeung
